# Development and psychometric evaluation of a new patient -reported outcome measure for stroke self -management: The Southampton Stroke Self - Management Questionnaire (SSSMQ)

**DOI:** 10.1186/s12955-015-0349-7

**Published:** 2015-10-03

**Authors:** Emma J Boger, Matthew Hankins, Sara H Demain, Susan M Latter

**Affiliations:** Faculty of Health Sciences, University of Southampton, Southampton, SO17 1BJ UK; Real-World Evidence Solutions, IMS Health, London, N1 9JY UK

**Keywords:** Stroke, Self-management, PROM development, Non-parametric item response theory, Reliability and validity

## Abstract

**Background:**

Self-management is important to the recovery and quality of life of people following stroke. Many interventions to support self-management following stroke have been developed, however to date no reliable and valid outcome measure exists to support their evaluation. This study outlines the development and preliminary investigation of the psychometric performance of a newly developed patient-reported outcome measure (PROM) of self-management competency following stroke; the Southampton Stroke Self-Management Questionnaire (SSSMQ).

**Methods:**

A convenience sample of 87 people who had had a stroke completed responses to the SSSMQ, the Stroke Self-Efficacy Questionnaire and the Stroke Impact Scale. Scaling properties were assessed using Mokken Scale Analysis. Reliability and construct validity were assessed using intra-class correlation coefficient (ICC), Mokken and Cronbach’s reliability coefficients and Spearman rank order correlations with relevant measures.

**Results:**

Mokken scaling refined the SSSMQ to 28 scalable items. Internal consistency reliability (Mokken *r* = 0.89) and test-retest reliability (ICC = 0.928) were excellent. Hypotheses of expected correlations with additional measures held, demonstrating good evidence for construct validity.

**Conclusions:**

Early findings suggest the Southampton Stroke Self-Management Questionnaire is a reliable and valid scale of self-management competency. The SSSMQ represents a potentially valid PROM for the evaluation of self-management following stroke.

## Background

Stroke is a major cause of death and disability world-wide [1] with potential long-term impact for the individual, such as social isolation [2, 3], long-term depression and anxiety [4, 5] and increased physical impairment [6]. Recovery following stroke is complex and multidimensional, encompassing bio-medical, psychological and sociological elements [7, 8]. Engagement in self-management practices by individuals with long-term conditions has been suggested as key to promoting recovery [9] and is cited as a means of empowerment and a facilitator of improved health outcomes [10, 11]. Self-management, defined as those activities (examples include eating well, exercising, taking medicines, monitoring and managing symptoms) people with a long-term health condition do to stay well and maintain good physical and emotional well-being [12] is not new to western health care. Since the development of the United States based chronic disease self-management programme [13] and subsequent adaption for other western health care settings [14–16], self-management has received growing attention as part of the management of people with long-term conditions [17, 18].

Self-management interventions (SMIs) are designed to enable people to manage their health more effectively. Stroke SMIs often aim to modify attitudes and behaviours such as goal-setting and lifestyle changes [19, 20] requiring the development or enhancement of skills to self-manage effectively. However, what elements should be included in stroke SMIs remains inconclusive [21]. Patient-Reported Outcome Measures (PROMs) measure how patients function or feel in relation to their health condition, and are recognised as fundamental health outcomes in their own right [22]. A systematic review identified that stroke SMIs have been evaluated by measurement of heterogeneous concepts such as physical function, quality of life, self-efficacy, satisfaction and mood (Boger et al. 2013). The review identified 43 different outcome measures, of which 21 measures (49 %) demonstrated some psychometric properties in stroke populations. This indicates a lack of consensus regarding the appropriate measures to assist evaluation of stroke SMIs, and/or a recognition that self-management embraces a range of differing concepts. The majority of measures scored either ‘fair’ or ‘poor’ according to the COSMIN quality criteria [23], possessing limited psychometric validity. For example, the Barthel Index [24] a measure of physical function, scored poorly due its lack of engagement with users in development and the Functional Independence Measure [25] a measure of physical and cognitive disability, scored ‘fair’ for internal consistency.

No outcome measure exists to adequately measure stroke self-management behaviours, attitudes or skills as discrete concepts, or from the patients’ perspective (Boger et al., 2013). This study used non-parametric item response theory (IRT) to investigate the psychometric properties of a new patient-reported outcome measure of stroke self-management competency to help address this knowledge gap and enhance the evidence quality in terms of the effectiveness of SMIs.

## Aims

To investigate the scale structure, reliability and evidence for validity of the Southampton Stroke Self-Management Questionnaire (SSSMQ).

## Methods

### Item elicitation

Items for the SSSMQ were developed following two qualitative phases involving people who had experienced a stroke, to enhance content validity and ensure item relevance [26, 27]. Five focus groups (*n* = 28) were first conducted to identify the factors which facilitate and hinder self-management following stroke, and to generate a preliminary item pool. The findings of this research have been reported elsewhere [28]. Cognitive interviewing, a technique to ensure items included in PROMs have precision and relevance to potential respondents (Willis, 2005), was then used to investigate the suitability, acceptability and interpretation of the item pool with an additional sample of people who had experienced stroke (*n* = 11). Preferences for response format were also investigated during cognitive interviewing. Participant’s feedback was sought on three different response scales; the six-point scale was chosen based on the preferences of the sample.

44 items were presented for psychometric testing (Appendix 1: Table 4). Items were rated on a 6 point Likert-style (Always true = 6, Always false = 1), based on the preferences of the cognitive interview sample for response format. Reverse scoring was applied on some items (3–5, 7, 9–13, 22, 24, 26, 28, 34, 41).

### Scale development

## Sample

A nationally recruited convenience sample was sought *via* advertisement on websites and newsletters of two UK-wide stroke charities, stroke support groups, selected from regions across the UK, and from a regional stroke research participant newsletter. Participants were community dwelling adults (18 years or over), who reported having at least one stroke not less than 3 months previously, who had been discharged from acute medical management and who could read English sufficiently to complete a questionnaire written in English. No upper age limits were applied. Participants were asked whether they had “ever taken part in a stroke or health education programme (run by health professionals or patient experts)”: none reported that they had done so.

Two additional patient-report outcome measures were used to investigate construct validity; the Stroke Impact Scale version 3.0 (SIS) [29] and the *Stroke Self-Efficacy Questionnaire* (SSEQ) [30, 31]. The SIS is a measure of health status after stroke and consists of 59 items, entailing eight domains as follows: Strength; Hand function; ADL/IADL; Mobility; Communication; Emotion; Memory and thinking and Participation/Role function. Responders rate the level of difficulty pertaining to each item on a 5-point Likert response format, generating summative scores for each domain. Internal consistency reliability ranges from α = 0.83–0.90 [32]. We hypothesised that health status would be positively associated with self-management skills, attitudes and behaviours. Hence, higher scores within each domain of the SIS would correlate with higher scores on the SSSMQ.

At the time the study was conducted, the SSEQ consisted of a 13-item self-report scale measuring self-efficacy judgements in specific domains of functioning post stroke using a 0–10 visual analogue response scale. Internal consistency reliability of the SSEQ is reported as α = 0.90 [30]. Following subsequent Rasch analysis [31], the SSEQ is thought to measure two distinct unidimensional constructs of self-management self-efficacy and activity self-efficacy, and be better suited to a four-point response scale. The SSEQ was selected to examine the hypothesis that stroke specific self-efficacy was positively associated with self-management skills, attitudes and behaviours. Hence higher scores on the SSEQ would correlate with higher scores on the SSSMQ.

Data regarding age, gender, length of time since most recent stroke, living situation and ethnicity were also collected *via* self-report.

Participants received an information sheet about the study and could opt to complete a paper (postage paid return envelope included), or an on-line version of the questionnaires (permission was gained from the authors of the SSEQ and SIS to convert them to an online format) using survey software (www.smartsurvey.co.uk). To avoid over-burdening participants during test-retest reliability evaluation, participants who completed the first questionnaire were invited to opt into completing a repeat SSSMQ (repeat testing of the other measures was not deemed necessary as these already have proven reliability.). To avoid a potential bias from this group, a sub-sample of those who opted to complete a second SSSMQ were randomly selected, two weeks after first completion, using block randomisation. Test-retest participants were grouped consecutively into a ‘block’ of five participants and randomised as follows:

Block 1 – YYNNY Block 2 – YNYNY Block 3- NNYYY

(Where, ‘Y’ received second mailing, ‘N’ did not receive second mailing).

Following the first 15 positive responders, the block allocation resumed at block 1, and repeated until close of recruitment.

### Data analysis

Data were coded and entered into SPSS 21.0 (Statistical Package Program for Social Sciences; SPSS Inc., Chicago, IL, USA) for the analysis of descriptive statistics, investigation of reliability and for correlations with additional measures. Data were saved in a tab-delimited format for import into the Mokken Scaling Procedure (MSP) [33] and scale structure was investigated using the Automated Item Selection Procedure (AISP) for Mokken Scale Analysis (MSA) within the ‘R’ statistical package ‘Mokken’ [34]. The Mokken package was used to test for items which produce monotonically non-decreasing item characteristic curves. Using the MSP, a unidimensional scale is conventionally indicated by a Loevinger’s coefficient (H) >0.3 [35]. Items violating monotone homogeneity were removed from the analysis until no further violations were present, ensuring a unidimensional scale.

The study received peer review and ethical and research governance approval prior to data collection commencing (University of Southampton, Faculty of Health Sciences Research Ethics committee, FoHS-2982). Following receipt of a participant information sheet, completion and return of the questionnaires *via* either mode indicated consent for participation in the study.

## Results

During April -June 2013, 87 completed responses were received from a total of 95 returned questionnaires. Six (postal) incomplete responses were returned and were excluded from the analysis. The majority of responses were submitted *via* the online method (62 %). Response rate for those completing online responses was not calculated due to difficulties in accurately assessing how many people had viewed *via* this medium. The response rate for those returning paper versions was 62 %. Respondent characteristics are summarised in Table 1.

**Table 1:** Summary of sample characteristics

Questionnaire			
	SSSMQ^a^	SSEQ^a^	SIS^a^
No. of complete responses (% of returned responses, *n* = 95)	87	83	74
	(92)	(87)	(78)
Age range (years)	(27–89)	(27–89)	(27–89)
Mean	58.40	59.20	57.99
[SD]	[14.74]	[14.74]	[14.66]
Male gender (%)	40 (51)	43 (52)	37 (50)
Mean months since	55	59	51
stroke [SD]	[63.36]	[63.82]	[56.29]
range#	319#	319#	273#
Mode of completion			
Paper (% of returned responses, *n* = 95)	26	26	23
	(38)	(38)	(24)
Online (% of returned responses, *n* = 95)	69	69	72
	(62)	(62)	(76)
Living situation			
Live with others	79	78	78
Live alone	19	21	22
Unknown	2	1	0
Ethnicity			
White European ethnicity	90	90	89
Non-white European ethnicity	10	10	11

^a^ *SSSMQ* Southampton Self-Management Questionnaire, *SSEQ* Stroke Self-Efficacy Questionnaire, *SIS* Stroke Impact Scale

Conceivably, those who live with others may possess a greater level of dependency due to impairment, which may have a bearing on self-management ability. In this sample, one way analysis of variance (ANOVA) tests revealed that perceived level of recovery (as measured by the SIS) was not statistically different for those who lived alone (mean = 64.0, SD = 26.4) compared to those who lived with partners or family members (mean = 54.2, SD = 21.8) (*p* = 0.06). Self-efficacy (Stroke Self-efficacy Questionnaire), was also not statistically significant between those who lived alone (mean 80.6, SD = 34) or with others (mean = 90.6, SD = 42.7) (*p* = 0.376).

### Scale determination

From the 44 items presented for scale determination, 12 items did not meet the criteria for a Mokken scale (*H* >0.3) and were thus excluded from the Mokken analysis. The remaining 32 items possessed individual *H* coefficients ranging from 0.96–0.39, with an overall *H* coefficient of 0.274, indicating a borderline Mokken scale [36]. Using the Automated Item Selection Procedure (AISP) for MSA within *R* statistical software package [34], threshold values were incrementally increased by .05 from > .35– > .5 for the remaining 32 items.

**Fig. 1:**
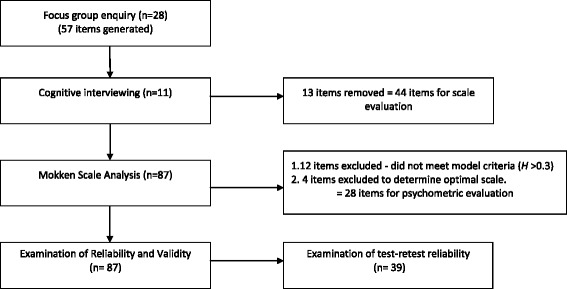
Summary of SSSMQ development

Items remained as one scale at the >0.35 threshold, but split as thresholds increased. Items retained at the >0.3, >0.35 and >0.4 thresholds were re-examined. Four items worded similarly to items retained in the scale and sharing the same *H* coefficient were removed, based on which item was best interpreted by potential users of the scale, informed by the cognitive interviewing data (appendix 1). Loevinger’s coefficient *H* for the resulting 28-itemed scale was 0.353, indicative of an acceptable Mokken scale. Figure 1 summarises the development phases.

For investigation of reliability and validity, scores from the 28-itemed scale were used. Internal consistency reliability of the 28-itemed SSSMQ was examined using Cronbach’s α [37] and Mokken’s estimation of reliability, denoted *r* [38]; values for α and *r* were both 0.89, indicating excellent internal consistency reliability [39]. MSA revealed the optimum number of items necessary to form a Mokken scale; there was no item redundancy. Test-retest reliability with a sub-sample of respondents (*n* = 39, 85 % response rate) using the intraclass correlation coefficient was 0.928 indicating a high degree of reliability of the scale [40]. Mean time between the first and second test was 14.51 days (SD 5.8). Mean age of the test-retest sample was 60.13 years (SD 14.28); 64 % of the sub-sample were male.

### Validity

Total scores from the refined 28-item SSSMQ, were moderately and in most cases, significantly associated, in expected directions with variables hypothesised to influence stroke self-management attitudes, behaviours and skills (Table 2) providing preliminary evidence of convergent validity. Mean item scores were broadly centrally spread (Table 3). Mean total scores on the SSSMQ were statistically significant according to gender (female mean = 118.0, SD = 17.4, *versus* male mean = 105, SD 28.0 *p* = .020). Mean total SSSMQ scores were not statistically different for living circumstances (live alone mean = 112.6, SD = 16.7, *versus* 113.8, SD = 21.3 *p* = .130), nor age (Spearman’s rho 0.063, *p* = .576).

**Table 2:** Non-parametric correlation matrix of SSSMQ with additional measures

	SSSMQ	SSEQ	Strength~	Hand function~	ADL/IADL~	Mobility~	Communication~	Emotion~	Memory & thinking~	Participation/role function~	0-100 perceived recovery ~
SSSMQ		.62^a^	.05	.10	.24^a^	.25^a^	.38^a^	.59^a^	.49^a^	.36^a^	.61^a^

*SSSMQ* Southampton Stroke Self-Management Questionnaire (*n* = 87), *SSEQ* Stroke self-efficacy questionnaire (*n* = 83) ~ domain of the Stroke Impact Scale (*n* = 74) ^a^Significant at the 0.01 level or lower

**Table 3:** Item response summaries for SSSMQ (*n*=87) ^a^values rounded to 2 decimal places

Item No	Item (r- denotes reverse score)	Mean^a^	SD^a^
1	The effects of stroke mean that I cannot manage my recovery and health (r)	4.23	1.54
2	When things do not go well with my stroke, it is hard to stay positive (r)	3.04	1.45
3	It is not up to me to decide what the best ways to manage my stroke are (r)	2.72	1.19
4	The physical effects of stroke mean that I cannot manage my health as I would like (r)	3.84	1.60
5	It is hard to be motivated to seek out solutions to problems relating to stroke (r)	3.25	1.47
6	I am not sure what signs or symptoms might mean my health is changing (r)	4.27	1.56
7	My problems with communication mean that I cannot manage my health as I would like (r)	3.92	1.68
8	Whatever I do, I will not improve my condition (r)	4.50	1.15
9	The efforts I take to manage my health have a positive effect	4.84	1.11
10	I find it difficult to tell health care professionals what I want or need (r)	4.83	1.27
11	I work out ways of managing my health following stroke together with health care professionals	3.68	1.41
12	I am confident that health care professionals can answer my questions	4.87	1.41
13	I feel confident at discussing any advice I don’t understand with Doctors	3.90	1.64
14	I feel confident at getting the information I need from health care professionals	3.50	1.58
15	I know how to get help if I am concerned about my condition	3.86	1.41
16	I plan my day so I can get things done without being tired	4.18	1.41
17	I feel confident asking family members to help me do things important to my health	4.08	1.29
18	I manage things related to stroke as well as other people with stroke	3.19	1.64
19	I try different ways of doing things, until I find out what works for me	4.24	1.66
20	Ideas and things that work for other people with stroke are helpful to my recovery	4.27	1.62
21	I have useful information or advice to give to others regarding managing stroke	3.83	1.57
22	I feel comfortable asking friends to help me do things important to my health	4.12	1.48
23	I am concerned that the things I do to manage stroke, may cause harm if not guided by health care professionals (r)	4.58	1.09
24	I cannot alter what my healthcare professionals decide to do about my stroke (r)	4.67	1.03
25	My condition would improve if I received more professional help (r)	4.55	1.23
26	Following advice from health care professionals is the only way I will manage stroke (r)	4.10	1.50
27	I always follow professional advice about my health, to the letter (r)	4.53	1.49
28	Constant professional advice would help me to manage my stroke (r)	4.56	1.03

## Discussion

Mokken scale analysis revealed that the SSSMQ forms an acceptable unidimensional scale consisting of 28 items. Each item concerns a stroke self-management attitude, skill or behaviour. Collectively, based on the preliminary evidence of validity reported here, and subject to further validation work, the SSSMQ measures stroke self-management competency, the features an individual requires to be competent and capable of managing health and wellbeing following stroke.

Self-efficacy is the most commonly purported theoretical basis underpinning stroke self-management interventions [21, 41], whereby individuals with higher self-efficacy are thought better able to self-manage [42]. Self-efficacy is thought to mediate desirable health behaviours, such as, following a healthy lifestyle, taking prescription medication, that lead to improved motivation, treatment adherence, function and better clinical outcomes [43, 44]. Lower levels of self-efficacy are associated with lower mood and coping skills after stroke [45]. Consistent with this literature, SSSMQ scores correlated moderately with stroke self-efficacy, as measured by the SSEQ (Table 2) [46]. Higher self-efficacy scores were associated with increased SSSMQ scores and thus more successful self-management behaviours, attitudes and skills.

Physical function is likely to affect individual’s ability to perform tasks or strategies important to self-management [47, 48] along with social, emotional and cognitive factors. Total scores from the SSSMQ moderately correlated with the Strength; Hand function; ADL/IADL and Mobility domains of the SIS, although correlations with the Hand function and Strength domains were weak and not significant (Table 2). Further investigation of the relationship between self-management competency and physical function is therefore required.

Better self-management is thought to lead to improved well-being and mood [47], significant to recovery following stroke [48]. Low mood has been identified as a barrier to self-management [49, 50]. Scores from the SSSMQ correlated positively with scores on the emotion domain of the SIS, suggesting that, individuals with lower mood, exhibit fewer desirable self-management behaviours and attitudes. Improved mood may augment self-management competency, or potentially *vice versa*. Positive correlations were also observed with the communication, memory and participation domains of the SIS, suggesting that these elements are important to self-management competency. Effective communication, which in stroke may be hindered by the presence of aphasia, is likely to enabled successful self-management [51] as navigating services and negotiating treatment strategies with professionals is key to self-management after stroke [52]. Improvement in the communication and participation domains of the SIS have been reported following a stroke self-management intervention [53], suggesting that effective self-management may have the potential to impact upon these domains.

Correlations with the SSSMQ and the SIS subscales provide preliminary evidence that successful self-management is associated with improved quality of life. Conceptually, quality of life may be important to self-management; gaining more control over health and well-being can feasibly be considered to improve quality of life as people develop the coping skills to adjust to and manage their life post-stroke [47, 54]. Alternatively, those who possess a greater quality of life may be more likely to exhibit the skills necessary to self-manage competently.

A difference between gender and total SSSMQ scores was found. It is not surprising that gender might impact upon self-management attitudes, behaviours and skills, as women typically perform better in self-management interventions [55–57]. Total SSSMQ scores were not statistically associated with age or living circumstances as might have been expected given that previous research indicates that older adults and people who live alone, often find self-management more difficult [50, 58, 59]. Further development of the SSSMQ with additional psychometric testing is warranted to provide continuing evidence of discriminant validity.

Investigation of reliability demonstrates that the SSSMQ is an adequately stable measurement of stroke self-management competency. Internal consistency and test-retest reliability were excellent, but must be considered in light of the limitations of sample size.

The findings provide preliminary evidence of the reliability and validity of the SSSMQ. The predicted hypotheses made with regard to the relationship of scores from the SSSMQ and additional measures were borne out, suggesting that self-management competency is consistent with previously validated measures of stroke self-management.

The optimal content, target outcomes, and mechanisms for change in stroke self-management interventions remain unclear [21, 60]. Measurement of an individuals’ self-management competency, their attitudes towards self-management and relevant behaviours relies upon patient report. The SSSMQ potentially represents an instrument, grounded in the views of patients who have had experienced stroke, with which to evaluate the impact of interventions on stroke self-management competency following stroke.

There are several limitations of the study which are acknowledged. There are no definitive answers regarding sample size requirements for IRT, however sample sizes of 100 are often adequate [61]. Therefore, the inferences drawn from the results must be considered in light of the relatively small sample size, and the possibility for type II errors in analysis. The average age of the UK stroke population is 75 years [62]. The sample in this UK based research was considerably younger at just over 58 years, which may in part explain the larger proportion of people who chose to take part on-line. Moreover, it is not possible to say if those taking part on-line had differing competency at using computers or different access to computers compared to a typical stroke population. The SSSMQ and study information were only available in English, which may have prevented or dissuaded those who do not have English as a first language from participating. Over 60 % of the sample had a moderate communication impairment, according to scores from the communication domain (<60) of the Stroke Impact Scale. This is a strength of this study since people with communication impairment are often excluded from stroke research.

It is also acknowledged that a tension potentially exists between the items, which were inductively generated and considered important to potential users, and the criteria for discarding items that do not function well in a scale [63]. Nonetheless, Mokken scaling represents a measurement model with the least criteria in this respect and is the method most likely to resolve this tension in favour of retaining items [64, 65].

Further investigation of validity, including cross-cultural applications, is necessary to provide further evidence of the psychometric properties of the SSSMQ with a larger, more diverse sample. Future studies should also include clinician/researcher obtained data regarding participants’ level of impairment. This would aid judgements about the relationship between impairment and self-management competency. In this research, selection of outcome measures with which to investigate theoretical relationships with the SSSMQ focused on the prevailing theory of self-efficacy. Further exploration of construct validity with additional measures of concepts associated with self-management, such as health literacy, decision-making and the burden of self-management may further enhance the strength of the construct of self-management competency.

## Conclusions

Mokken scale analysis revealed a 28-itemed outcome measure with acceptable scaling properties which can potentially be used to enhance evaluation of stroke self-management interventions in research and clinical practice. Early findings suggest that the SSSMQ possesses excellent reliability and preliminary evidence of validity. Further investigation of validity and reliability of the SSSMQ is required. It follows that totalled item scores from the SSSMQ can potentially be used as an indicator of an individuals’ level of self-management competency.

## Appendix 1

**Table 4:** Full items presented for psychometric testing

**Item NO**	**Item**
1	*I regularly think about how I might change things to better manage the consequences of stroke*
2	*I cannot rely solely on others to help me manage the consequences of stroke*
3	**Whatever I do, I will not improve my condition (r)**
4	**When things do not go well with my stroke, it is hard to stay positive (r)**
5	**I always follow professional advice about my health, to the letter (r)**
6	*General self-care is important to me to stay as healthy as possible*
7	There is little point in trying new ways of managing the consequences of stroke, as it will not change my condition (r)
8	*I should make most of the decisions about how my stroke is managed*
9	**It is hard to be motivated to seek out solutions to problems relating to stroke (r)**
10	It is difficult to stay motivated to do tasks or strategies important to my recovery (r)
11	**My condition would improve if I received more professional help (r)**
12	**It is not up to me to decide what the best ways to manage my stroke are (r)**
13	**I cannot alter what my healthcare professionals decide to do about my stroke (r)**
14	*Treatment or therapy regimes do not take over my life*
15	*I apply professional advice so it is relevant to my situation*
16	**I plan my day so I can get things done without being tired**
17	*I talk about any medication I am prescribed with healthcare professionals*
18	*I find answers to problems about stroke without seeking professional advice*
19	I try different ways of doing things, until I find out what works for my health
20	**I know how to get help if I am concerned about my condition**
21	*The lifestyle choices that affect my health (for example, smoking, diet, alcohol,* etc.*) have changed since having a stroke*
22	**I am concerned that the things I do to manage stroke, may cause harm if not guided by health care professionals (r)**
23	**I feel confident at discussing any advice I don’t understand with Doctors**
24	**I find it difficult to tell health care professionals what I want or need (r)**
25	*I always ask health care professionals to explain why I should follow their advice*
26	**Constant professional advice would help me to manage my stroke (r)**
27	**I am confident that health care professionals can answer my questions**
28	**Following advice from health care professionals is the only way I will manage stroke (r)**
29	**I work out ways of managing my health following stroke together with health care professionals**
30	**The physical effects of stroke mean that I cannot manage my health as I would like (r)**
31	**My problems with communication mean that I cannot manage my health as I would like (r)**
32	*The effects of stroke mean that I cannot control any aspect of my recovery and health*
33	**The effects of stroke mean that I cannot manage my recovery and health (r)**
34	**I am not sure what signs or symptoms might mean my health is changing (r)**
35	**I feel confident at getting the information I need from health care professionals**
36	My efforts to manage the consequences of stroke turn out how I like
37	I am able to control my general health
38	**The efforts I take to manage my health have a positive effect**
39	**I manage things related to stroke as well as other people with stroke**
40	**I have useful information or advice to give to others regarding managing stroke**
41	*I am reliant upon others to help me do things important to my health and well-being (this might for example, involve attending appointments, shopping for food, social activities) (r)*
42	**I feel comfortable asking friends to help me do things important to my health**
43	**I feel confident asking family members to help me do things important to my health**
44	**Ideas and things that work for other people with stroke are helpful to my recovery**

Items in BOLD text were retained following analysis (*r*) = indicate items that are reverse scored Items in *italics* did not meet model criteria (H coefficients <0.3)
